# P-880. Risk Factors of In-Hospital Mortality among Adult Patients with *Burkholderia cepacia* Complex Bacteremia Admitted to the Philippine General Hospital: A Retrospective Cohort Study

**DOI:** 10.1093/ofid/ofae631.1071

**Published:** 2025-01-29

**Authors:** Sergie Paul Christoffer C Fernandez, Maria Sonia Salamat

**Affiliations:** Philippine General Hospital, Manila, National Capital Region, Philippines; Philippine General Hospital, Manila, National Capital Region, Philippines

## Abstract

**Background:**

Burkholderia cepacia complex (BCC) has emerged as a significant nosocomial pathogen, particularly in immunocompromised patients, leading to various infections with a high mortality rate. There is limited data on BCC bacteremia in the Philippines. Hence, more study is necessary to enhance patient management and outcomes.

This study aims to identify significant risk factors for in-hospital mortality among adult patients with BCC bacteremia at the Philippine General Hospital. Additionally, it seeks to describe clinical characteristics, common foci of infection, and antimicrobial susceptibility patterns of BCC.

**Methods:**

A retrospective cohort study included adult patients admitted to the Philippine General Hospital with documented BCC bacteremia from January 2020 to December 2023. Demographic and clinical data were extracted from medical records, and BCC identification and resistance patterns were determined using the VITEK 2 system and MALDI-TOF technology. Statistical analyses were performed to identify mortality risk factors.

**Results:**

Out of 172 patient charts reviewed, 171 cases were included in the final analysis. Most patients were male, with a mean age of 52.7 years. Central-line associated bloodstream infections (CLABSI) were the most common source of bacteremia. Approximately 65% of cases received inappropriate empirical therapy. Resistance was highest for Meropenem (13.5%), followed by Co-trimoxazole (11.8%), while Ceftazidime and Minocycline showed lower resistance rates. All-cause mortality was 22.2%, with BCC bacteremia accounting for 57.9% of these deaths. Male gender, septic shock, mechanical ventilation, and prolonged duration of ineffective antimicrobial therapy were the significant risk factors for all-cause mortality.

**Conclusion:**

The study revealed a significant in-hospital mortality rate associated with BCC bacteremia, predominantly caused by CLABSI and compounded by high antimicrobial resistance and inappropriate empirical therapy. These findings underscored the need for accurate microbiological diagnostics, appropriate antibiotic usage, and robust infection control practices to improve patient outcomes.

**Disclosures:**

**All Authors**: No reported disclosures

